# Glycyrrhetic Acid Ameliorates Dextran Sulfate Sodium-Induced Ulcerative Colitis *in Vivo*

**DOI:** 10.3390/molecules21040523

**Published:** 2016-04-22

**Authors:** Yong-Deok Jeon, Sa-Haeng Kang, Keuk-Soo Bang, Young-Nam Chang, Jong-Hyun Lee, Jong-Sik Jin

**Affiliations:** 1 Department of Oriental Medicine Resources, Chonbuk National University, 79 Gobongro, Iksan 570-752(1), Korea; dugicom@nate.com (Y.-D.J.); rkdtkgod@naver.com (S.-H.K.); ksbang@jbnu.ac.kr (K.-S.B.); chyn@jbnu.ac.kr (Y.-N.C.); 2 Department of Pharmacy, College of Pharmacy, Dongduk Woman’s University, 23-1 Wolgok-Dong, SungBuk-Gu, Seoul 136-714, Korea; naturalmed@dongduk.ac.kr

**Keywords:** glycyrrhetic acid, ulcerative colitis, dextran sulfate sodium, anti-inflammation

## Abstract

Glycyrrhizae Radix (GR) is a Korean traditional herb medicine that is widely used in clinical health care. Glycyrrhetic acid (GA) is an aglycone saponin extracted from GR that has anti-inflammatory, anti-cancer, and anti-viral effects. However, the anti-inflammatory effects of GA in colitis have not been reported. This study investigated the role of GA on ulcerative colitis in a dextran sulfate sodium (DSS)-induced mouse colitis model. DSS-treated mice displayed weight loss and shortened colon length compared with control mice. Mice administered GA showed less weight loss and longer colon length than the DSS-treated group. Interleukin (IL)-6, IL-1β, and tumor necrosis factor-alpha were decreased by GA treatment. GA treatment also reduced DSS-induced microscopic damage to colon tissue. GA regulates the phosphorylation of transcription factors including nuclear factor-kappa B (NF-κB) and IκB alpha, and regulates the expression of cycloxygenase-2 and prostaglandin E_2_. GA thus showed beneficial effects in a mouse model of colitis, implicating GA might be a useful herb-derived medicine in the treatment of ulcerative colitis.

## 1. Introduction

Ulcerative colitis (UC) is a chronic and relapsing inflammatory disease characterized by dysregulation of the immune function response and imbalanced release of cytokines and unresolved inflammatory progress associated with intestinal mucosa [1,2]. The inflammation reaction may be initiated by pro-inflammatory cytokines [3]. Patients with bowel disease have increased interleukin (IL)-6 in the intestinal mucosa and tumor necrosis factor-alpha (TNF-α) in the blood and colon tissue. Immune cells, such as T cells, intestinal epithelial cells, and macrophages, secrete various cytokines including TNF-α, IL-1 family, IL-6, IL-8, and interferon-gamma (IFN-γ), which regulate the inflammatory response in UC. Elevated levels of cytokines have also been implicated in the pathogenesis of bowel disease [4,5].

Dextran sulfate sodium (DSS)-induced colitis is considered as a suitable model that represents the disease both morphologically and biochemically. Intake of DSS induces bloody stools, ulcerations, epithelial injury, and infiltration of inflammatory cells [6]. Histologically, DSS causes crypt abscesses and epithelioglandular hyperplasia in mice. These colon conditions are similar to acute and chronic UC in humans. DSS is toxic to gut epithelial cells of the basal crypts and affects the integrity of the mucosal barrier [7]. Thus, the DSS-induced colitis model is especially suitable to study the mechanism of inflammatory colitis.

Cyclooxygenase-1 (COX-1) is a constitutive enzyme that catalyzes the production of prostaglandins (PGs), which protect the stomach from damage. COX-2 is induced by inflammatory stimuli, such as cytokines, and also catalyzes PG production that contributes to inflammation-related swelling [8]. COX-2 and PGE2 levels are raised in the inflamed mucosa of patients with UC [9].

*Glycyrrhiza uralensis* (GU) is a medicinal plant used in Europe and Asia for treating debilitating UC. It has been also used for diseases of the respiratory system and gastrointestinal tract [10]. Glycyrrhizae Radix (GR) has been traditionally used for treatment of cough, allergy, and bowel disease [11]. GR possesses various bioactive constituents that include saponins, flavonoids, and coumarins [12]. However, it is unknown whether the constituents of GR can regulate intestinal inflammation. Glycyrrhetic acid (GA) is a triterpenoid saponin found in GU as a hydrolyzed metabolite of glycyrrhizin [13]. Recent studies have demonstrated that GA has strong antioxidant, anti-inflammation, and anti-cancer effects [14]. GA can reduce the oxidative stress caused by carbon tetrachloride, reduces production of pro-inflammatory cytokines, and repress immune function [15]. These results suggest that GA has inhibitory effects on DSS-induced colitis model.

To explore the potential of GA as a useful therapeutic in UC, we examined its effects on DSS-induced UC in a mouse model. The aims were to evaluate the effect of GA on clinical signs including weight loss, colon shortening, diarrhea, and gross bleeding, and to investigate the role of GA on inflammatory mediators in DSS-treated mice.

## 2. Results

### 2.1. Effects of GA on Clinical Signs in DSS-Induced Colitis

**Figure 1:**
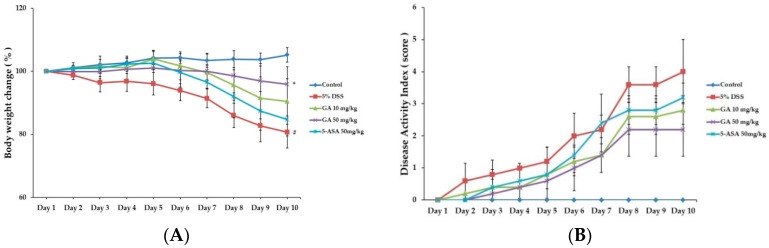
Effects of GA on clinical signs in DSS-induced colitis. (**A**) Body weight was measured at the same time on the experimental days; (**B**) Disease activity index score in the five study groups. Values represent mean ± S.E.M. (*n* = 6). Data were analyzed by Student’s *t*-test (^#^ *p* < 0.05 *vs.* control and * *p* < 0.05 *vs.* DSS alone).

**Figure 2:**
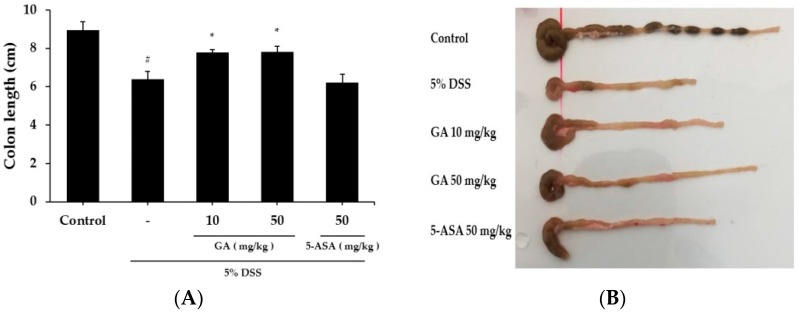
Effects of GA on DSS-induced colon shortening. (**A**) Average colon length in cm measured after 10 days at the time of sacrifice; (**B**) Representative colons of each group. Values represent mean ± S.E.M. (*n* = 6). Data were analyzed by Student’s *t*-test (^#^ *p* < 0.05 *vs.* control and * *p* < 0.05 *vs.* DSS alone).

When mice were treated with DSS to induce colitis, body weight was reduced and colon length was shortened at day 10 by 19.02 ± 1.51 g and 28.6% respectively, compared to the control group (Figure 1A and Figure 2A,B). Both GA 10 and GA 50 mg/kg alleviated the effects of DSS on body weight loss by 20.86 ± 1.76 g and 22.05 ± 1.25 g, and colon shortening by 13.2% and 12.7%, respectively (Figure 1B). Disease activity index (DAI) scores were increased at day 10 by 4.0 ± 1.00 in DSS-treated group, compared to the control group. GA also attenuated the DSS-mediated increase in DAI scores at day 10 by 2.8 ± 0.45, 2.2 ± 0.84; GA 10, GA 50 mg/kg.

### 2.2. Effects of GA on Inflammatory Cytokines Level in DSS-Induced UC

**Figure 3:**
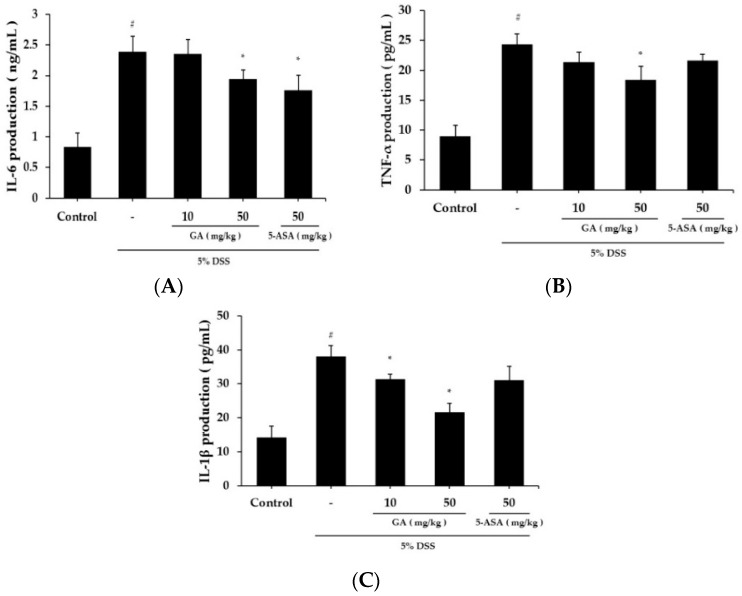
Effects of inflammatory cytokines levels in DSS-induced UC. (**A**) IL-6 production in mouse serum at day 10; (**B**) TNF-α production in mouse serum at day 10; (**C**) IL-1β production in mouse serum at day 10. Values represent mean ± S.E.M. (*n* = 6). Data were analyzed by Student’s *t*-test (^#^ *p* < 0.05 *vs.* control and * *p* < 0.05 *vs.* DSS alone).

Serum IL-6 level was significantly higher in the DSS treatment group (2.394 ± 0.246 ng/mL) than in the control group (0.839 ± 0.227 ng/mL) (Figure 3A). IL-6 level was lower in the GA 50 mg/kg group (1.943 ± 0.152 ng/mL) than the DSS-treated group. The serum TNF-α level was also increased in the DSS treatment group (24.376 ± 1.76 pg/mL) compared to the control group (8.943 ± 1.847 pg/mL), but was significantly inhibited in the GA 50 mg/kg group (18.441 ± 2.246 pg/mL) (Figure 3B). The serum IL-1β level was increased in the DSS treatment group (38.105 ± 3.152 pg/mL) compared to the control group (14.318 ± 3.206 pg/mL) (Figure 3C). IL-1β levels were lower in GA treatment groups (GA 10 mg/kg; 31.416 ± 1.406 pg/mL, GA 50 mg/kg; 21.721 ± 2.548 pg/mL).

### 2.3. Effects of GA on Transcription Factors in DSS-Induced UC

**Figure 4:**
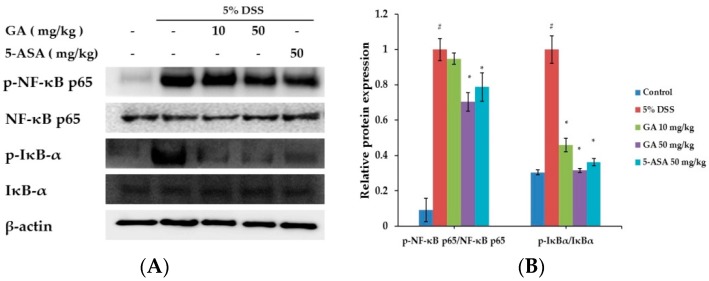
Effects of GA on transcription factors in DSS-induced UC. (**A**) Phosphorylation of IκBα and phosphorylation of NF-κB were assayed by Western blot; (**B**) Relative ratio of phospho-IκBα and NF-κB p65 calculated using an image analyzer. Values represent mean ± S.E.M. (*n* = 6). Data were analyzed by Student’s *t*-test (^#^ *p* < 0.05 *vs.* control and * *p* < 0.05 *vs.* DSS alone).

Nuclear factor-kappa B (NF-κB) is an important transcription factor of inflammation reactions [16]. The effect of GA on the activation of NF-κB was investigated (Figure 4). Phosphorylation of IκBα and NF-κB in the colon tissue was inhibited by GA treatment. These results suggested that GA can inhibit the activation of transcription factors in UC.

### 2.4. Effects of GA on Epithelial Injury in DSS-Induced UC

**Figure 5:**
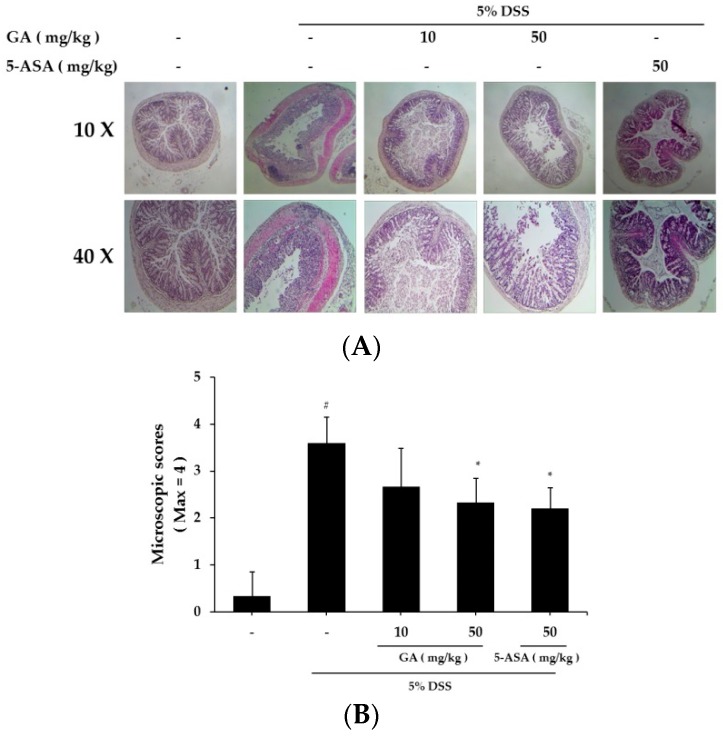
Effects of GA on epithelial injury in DSS-induced UC. (**A**) Paraffin sections of colonic tissue were stained with hematoxylin & eosin, and then observed by microscope (10× and 40×); (**B**) Microscopic scores. Values represent mean ± S.E.M. (*n* = 6). Data were analyzed by Student’s *t*-test (^#^ *p* < 0.05 *vs.* control and * *p* < 0.05 *vs.* DSS alone).

Mucosal thickness is regarded as an indicator of normal mucosal condition. DSS causes epithelial injury and infiltration of inflammatory cells, including mast cells [17]. We compared the colon tissue condition between the DSS-treated and control groups (Figure 5A). GA treatment attenuated the effects induced by DSS treatment (Figure 5B). GA treatment also reduced the DSS-mediated microscopic damage to the colonic tissue.

### 2.5. Effects of GA on COX-2 and PGE_2_ Expression in DSS-Induced UC

**Figure 6:**
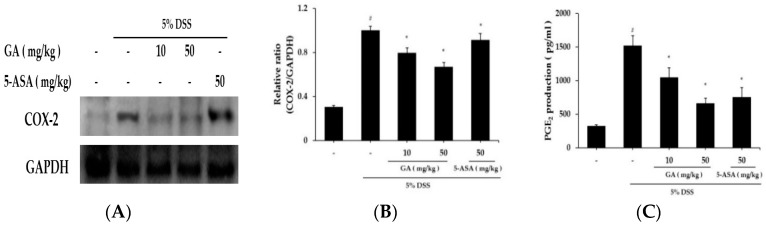
Effects of GA on COX-2 and PGE_2_ expression in DSS-induced UC. COX-2 levels were determined by western blot analysis, and PGE_2_ levels using PGE_2_ assay kits. (**A**) Western blot analysis was used to determine COX-2 levels in colonic tissues. Data shown are representative of three independent experiments; (**B**) COX-2/GAPDH ratios were determined by densitometry; (**C**) PGE_2_ production in colonic tissues. Values represent mean ± S.E.M. (*n* = 6). Data were analyzed by Student’s *t*-test (^#^ *p* < 0.05 *vs.* control and * *p* < 0.05 *vs.* DSS alone).

The effects of GA on COX-2 expression in colon tissues were demonstrated using western blot analysis (Figure 6A). DSS markedly induced COX-2 expression in colon tissue *vs.* control group, but increased COX-2 expression was significantly reduced by GA administration. Relative expression levels of COX-2 are presented in Figure 6B. COX-2 catalyzed PGE_2_ biosynthesis, and we examined whether GA affects PGE_2_ levels (Figure 6C). PGE_2_ levels were enhanced by DSS, and this increase was significantly inhibited by GA administration.

## 3. Discussion

Inflammatory bowel diseases (IBDs), including Crohn’s disease and UC, are chronic relapsing intestinal inflammatory disorders. Even if the pathogenic mechanism of IBDs is barely understood, recent evidence suggests that deviant immune responses cause the IBD symptoms [18,19,20,21]. UC symptoms can include weight loss and bloody diarrhea [22]. Immune modulators, such as sulfasalazine and glucocorticosteroids, are the most used therapies for UC [23]. These therapies can cause adverse side effects like vomiting, anemia, and generalized edema. Thus, interest in the use of traditional herbal medicines for inflammatory chronic disease has grown [24].

There are over the 20 animal models of colitis. DSS-induced colitis is one of the most suitable experimental models [25]. Using this model, we demonstrate herein that GA alleviates clinical signs of UC (weight loss, colon shortening, diarrhea and occult/gross bleeding).

IL-6, TNF-α, and IFN-γ are pathogenic mediators of murine colitis [26,27]. In IBD, immune cells including T lymphocytes and macrophages secrete inflammatory cytokines in the area of inflammation. These activated cells modulate the balance between pro- and anti-inflammatory cytokines. IL-6 and TNF-α expression is elevated in the rectal mucosa of UC patients [28]. Presently, GA suppressed the DSS-induced increase in IL-6, TNF-α, and IL-1β in mouse serum and tissues.

In the inflammation reaction, COX-1 protein activity rarely changes, but COX-2 production dramatically increases, leading to the increased production of PGs [29]. Among the PGs, PGE_2_ is increased in the intestines of IBD patients [30]. 5*-*Aminosalicylic acid is used to treat IBD by inhibiting COX-2 activation [31]. COX-2 and PGE_2_ play key role as mediators in UC. Presently, GA inhibited the DSS-induced activation of COX-2 and PGE_2_. The results suggest that the anti-inflammatory effect of GA is attributable to the regulation of COX-2 in DSS-induces colitis. We also found that GA reduced epithelial injury and inflammatory cell infiltration into colon tissue.

Pro-inflammatory mediators, such as inflammatory cytokines and chemokines, are controlled by the activity of transcriptional factors. The activation of NF-κB is important in the pathogenesis of IBD [32,33,34]. DSS-induced IκBα phosphorylation and phosphorylation of NF-κB p65 in colonic tissues were suppressed by GA treatment.

GR is used as a traditional herbal medicine in many Asian countries. Glycyrrhizin is the principal component of GR, and has been used for treatment of liver disease, allergic reactions, and gastric ulcers [35]. GA is a GR component which has an anti-inflammatory effect on lipopolysaccharide-induced liver injury and inhibition of hepatic lipotoxicity [36]. GA is a biologically active metabolite resulting from glycyrrhizin’s presystemic hydrolysis. Although GA has a protective effect on hepatic injury and an anti-inflammatory effect on LPS-stimulated macrophages [37], its regulatory effects on intestine inflammation have not been reported. GA is not cytotoxic to RAW264.7 macrophages in various concentrations [38]. GA also regulates nitric oxide generation, phosphorylation of mitogen activated protein kinaes (MAPKs), and NF-κB activation in infected bone marrow-derived macrophages [39]. These compounds might be useful for the prevention of inflammatory disease.

In summary, GA has anti-inflammatory effects in a DSS-induced colitis model. GA might inhibit inflammation by regulating the mediators like COX-2 and NF-κB. GA could be used for therapeutic agent to treat colitis.

## 4. Materials and Methods

### 4.1. Reagents

GA was purchased from Tokyo Chemical Industry CO (Tokyo, Japan). DSS was purchased from MP Bio (Santa Ana, CA, USA). 5-Aminosalicylic acid, 10% neutral-buffer, and eosin were purchased from Sigma-Aldrich (St. Louis, MO, USA). Purified, biotin, standard anti-mouse IL-6 and TNF-α, and (2,2′-azinobis [3-ethylbenzothiazoline-6-sulfonic acid] diammonium salt) substrate were purchased from BD Bioscience (San Diego, CA, USA). Lysis buffer was purchased from iNtRON Biotech (Seongnam, Gyeonggi-do, Korea). COX-2, NF-κB p65, pNF-κB p65, IκBα, and pIκBα were purchased from Cell Signaling Technology (Boston, MA, USA). Glyceraldehyde phosphate dehydrogenase (GAPDH) was purchased from Santa Cruz Biotechnology (Santa Cruz, CA, USA). Hematoxylin was purchased from Muto Pure Chemicals (Hongo, Bunkyo-ku, Tokyo, Japan).

### 4.2. Mice

Male BALB/c, 6-week-old mice obtained from SAMTACO (Osan, Kyungki-Do, Korea) were acclimatized in a specific pathogen-free environment under controlled conditions (22 ± 2 °C with a 12 h light/dark cycle) for one week. The mice were housed in five colony cages with six mice per cage.

### 4.3. DSS-Induced UC

All experimental protocols (CBU2016-0021) were approved by the Committee on the Care of Laboratory Animal Resources, Chonbuk National University and were conducted in accordance with the Guide for the Care and Use of Laboratory Animals. Acute colitis was induced by administering drinking water containing 5% (*w*/*v*) DSS to mice for 10 days. Thirty mice were weighted before the experiment, and were divided into five groups with six mice per group. Group 1 comprised untreated mice. Mice in group 2 received DSS. In group 3 and group 4, mice with induced colitis were treated with GA at 50 and 10 mg/kg, respectively. In group 5, mice with induced colitis were treated with 50 mg/kg 5-aminosalicylic acid as the reference drug. Mice were checked daily for body weight, stool consistency, and the presence of gross bleeding. GA was diluted with purified water and orally administered once a day during the 10 days of DSS treatment, after which the mice were sacrificed.

### 4.4. Disease Activity Index (DAI)

Intestinal disease activity was assessed based on the weight loss, presence of diarrhea accompanied by blood and mucus, and colonic shortening [40]. DAIs were calculated by scoring weight loss, diarrhea, and rectal bleeding based on the previously described scoring system [41] shown in Table 1. Weight loss was defined as the difference between initial and final weights, and diarrhea as the absence of fecal pellet formation and the presence of continuous fluid fecal material in the colon. Rectal bleeding was assessed based on the presence of diarrhea containing visible blood and on the presence of gross rectal bleeding. DAI values were calculated as {(weight loss score) + (diarrhea score) + (rectal bleeding score)}/4. The DAI was determined by three investigators blinded to the protocol. The clinical parameters used in the present study were chosen to represent the subjective clinical symptoms observed in human UC.

### 4.5. Enzyme-Linked Immunosorbent Assay (ELISA)

Levels of IL-6 and TNF-α in the serum and tissue were measured using an enzyme-linked immunosorbent assay (ELISA), as previously described [42]. Briefly, 96-well plates (SPL Life Science, Seoul, Korea) were coated with 100 μL of anti-mouse monoclonal antibody (1.0 mg/mL at pH 7.4 in phosphate buffered saline [PBS]) and incubated overnight at 4 °C. After additional washes, 50 μL of sample, or IL-6 and TNF-α standard was added and incubated at room temperature for 2 h. Plates were washed and 0.2 μg/mL of biotinylated anti-mouse antibody was added and incubated at room temperature for 2 h. After washing, avidin-peroxidase was added and plates were incubated for 30 min at 37 °C. The plates were then washed again and (2,2′-azinobis [3-ethylbenzothiazoline-6-sulfonic acid]-diammonium salt) substrate was added. Color development was measured at 405 nm using an automated microplate ELISA reader (Molecular Devices, Sunnyvale, CA, USA). Standard curves were prepared using serial dilutions of recombinant antibody. Protein concentrations were measured using bicinchoninic acid (BCA) protein assay reagent (Bio-Rad, Hercules, CA, USA).

### 4.6. Western Blot Analysis

Distal colons were homogenized in lysis buffer (iNtRON Biotech, Korea) and centrifuged at 13,000 rpm for 5 min. The supernatants were transferred to fresh tubes and protein concentrations were determined using BCA protein assay reagent. Lysates (50 μg protein) were separated by 10% SDS-PAGE and transferred to membranes (Amersham Pharmacia Biotech, Piscataway, NJ, USA), which were blocked with 5% skim milk in PBS-Tween-20 (PBST) for 1 h at room temperature. Membranes were incubated overnight with primary antibodies against COX-2, NF-κB p65, pNF-κB p65, IκBα and pIκBα and GAPDH, and washed three times with PBST. Blots were incubated with secondary antibody for 1 h at room temperature. Antibody-specific proteins were visualized using an enhanced chemiluminescence detection system (Amersham, Newark, NJ, USA). Protein densities were quantified by densitometry.

### 4.7. Histological Processing

Mice were sacrificed at the end of the experiment. The entire colon was dissected and flushed with ice-cold PBS. Sections of rectums were taken and fixed in 10% neutral-buffered formalin (Sigma-Aldrich) for 24 h at room temperature and embedded in paraffin to provide sections for histological evaluation. Severity of colitis was evaluated in sections stained with hematoxylin and eosin by two independent observers blinded to the experimental conditions according to modified criteria [43] summarized in Table 2.

### 4.8. PGE_2_ Assay

Distal colons were homogenized in lysis buffer, and centrifuged at 13,000 rpm for 5 min. PGE_2_ levels were quantified using immunoassay kits according to the manufacturer’s instructions (Enzo Life Science, East Farmingdale, NY, USA).

### 4.9. Statistical Analysis

The results are presented as mean ± S.E.M of at least three independent experiments. Results were analyzed using PASW Statistics 18.0 program. Student’s *t*-test was used to determine statistically significant differences. *p* < 0.05 was considered significant.

## 5. Conclusions

GA reduces colon length shortening and loss body weight by DSS treatment. GA regulates the levels of inflammatory mediators such as IL-6, TNF-α, and IL-1β in DSS-induced colitis in mice. GA treatment suppresses PGE_2_ production. It also reduces epithelial injury in DSS-induced UC. This study provides experimental evidence to show that GA might be a useful therapy in the treatment of UC.

## Figures and Tables

**Table 1:** Criteria for disease activity index.

Score	Weight Loss (%)	Stool Consistency	Bloodstain or Gross Bleeding
0	None	Normal	Negative
1	1–5	Loose stool	Negative
2	5–10	Loose stool	Positive
3	10–15	Diarrhea	Positive
4	>15	Diarrhea	Gross bleeding

**Table 2:** Criteria for assessment of microscopic rectal damage.

Score	Remarks
0	Normal colonic mucosa
1	Loss of one-third of the crypts
2	Loss of two-third of the crypts
3	Lamina propria covered with single layer of epithelial cells with mild inflammatory cell infiltration
4	Erosions and marked inflammatory cell infiltration

Mucosal damage was scored as 0–4 based on the loss of crypt (mucosa) and infiltration of inflammatory cells (maximum score = 4).

## References

[1] Arita M., Yoshida M., Hong S., Tjonahen E., Glickman J.N., Petasis N.A., Blumberg R.S., Serhan C.N., Resolvin E. (2005). Anendogenous lipid mediator derived from omega-3 eicopentanoic acid, protects against 2,4,6-trini-trobenzene sulfonic acid-induced colitis. PNAS.

[2] Libby P. (2002). Inflammation in atherosclerosis. Nature.

[3] Li Y., de Haar C., Chen M., Deuring J., Gerrits M.M., Smits R., Xia B., Kuipers E.J., van der Woude C.J. (2010). Disease-related expression of the IL6/STAT3/SOCS3 signaling pathway in ulcerative colitis and ulcerative colitis-related carcinogenesis. Gut.

[4] Ogata H., Hibi T. (2003). Cytokine and anti-cytokine therapies for inflammatory bowel disease. Curr. Pharm. Des..

[5] Papadakis K.A., Targan S.R. (2000). Role of cytokines in the pathogenesis of inflammatory bowel disease. Annu. Rev. Med..

[6] Cooper H.S., Murthy S.N., Shah R.S., Sedergran D.J. (1993). Clinicopathologic study of dextran sulfate sodium experimental murine colitis. Lab. Investig..

[7] Okayasu I., Hatakeyama S., Yamada M., Ohkusa T., Inagaki Y., Nakaya R. (1990). A novel method in the induction of reliable experimental acute and chronic ulcerative colitis in mice. Gastroenterology.

[8] Roberts P.J., Morgan K., Miller R., Hunter J.O., Middleton S.J. (2001). Neuronal COX-2 expression in human myenteric plexus in active inflammatory bowel disease. Gut.

[9] MacDonald T.T., Murch S.H. (1994). Aetiology and pathogenesis of chronic inflammatory bowel disease. Baillieres Clin. Gastroenterol..

[10] Asl M.N., Hosseinzadeh H. (2008). Review of pharmacological effects of *Glycyrrhiza* sp. and its bioactive compounds. Phytother. Res..

[11] Chang Y.I., Chen C.L., Kuo C.L., Chen B.C., You J.S. (2010). Glycyrrhetinic acid inhibits ICAM-1 expression via blocking JNK and NF-κB pathways in TNF-α-activated endothelial cells. Acta Pharmacol. Sin..

[12] Wang W., Luo M., Fu Y., Wang S., Efferth T., Zu Y. (2013). Glycyrrhizic acid nanoparticles inhibit LPS-induced inflammatory mediators in 264.7 mouse macrophages compared with unprocessed glycyrrhizic acid. Int. J. Nanomed..

[13] Kim Y.J., Lee C.S. (2008). Glycyrrhizin attenuates MPTP neurotoxicity in mouse and MPP-induced cell death in PC12 cells. Korean J. Physiol. Pharmacol..

[14] Matsui S., Matsumoto H., Sonoda Y., Ando K., Aizu-Yokota E., Sato T., Kasahara T. (2004). Glycyrrhizin and related compounds down-regulate production of inflammatory chemokines IL-8 and eotaxin 1 in a human lung fibroblast cell line. Int. Immunopharmacol..

[15] Agarwal M.K., Iqbal M., Athar M. (2005). Inhibitory effect of 18β-glycyrrhetinic acid on 12-*O*-tetradecanoyl phorbol-13-acetate-induced cutaneous oxidative stress and tumor promotion in mice. Redox Rep..

[16] Barnes P.J., Karin M. (1997). Nuclear factor-κB: A pivotal transcription factor in chronic inflammatory diseases. N. Engl. J. Med..

[17] Kim D.S., Ko J.H., Jeon Y.D., Han Y.H., Kim H.J., Poudel A., Jung H.J., Ku S.K., Park S.H., Park J.H. (2013). *Ixeris dentate* NAKAI reduces clinical score and HIF-1 expression in experimental colitis in mice. Evid. Based Complement. Altern. Med..

[18] Dharmani P., Chadee K. (2008). Biologic therapies against inflammatory bowel disease: A dysregulated immune system and the cross talk with gastrointestinal mucosa hold the key. Curr. Mol. Pharmacol..

[19] Strober W., Fuss I., Mannon P. (2007). The fundamental basis of inflammatory bowel disease. J. Clin. Investig..

[20] Sartor R.B. (2008). Microbial influences in inflammatory bowel diseases. Gastroenterology.

[21] Podolsky D.K. (2002). Inflammatory bowel disease. N. Engl. J. Med..

[22] Rufo P.A., Bousvaros A. (2006). Current therapy of inflammatory bowel disease in children. Paediatr. Drugs.

[23] Ishiguro Y., Ohkawara T., Sakuraba H., Yamagata K., Hiraga H., Yamaguchi S., Fukuda S., Munakata A., Nakane A., Nishihira J. (2006). Macrophage migration inhibitory factor has a proinflammatory activity via the p38 pathway in glucocorticoid-resistant ulcerative colitis. Clin. Immunol..

[24] Sandborn W.J., Targan S.R. (2002). Biologic therapy of inflammatory bowel disease. Gastroenterology.

[25] Wirtz S., Neufert C., Weigmann B., Neurath M.F. (2007). Chemically induced mouse models of intestinal inflammation. Nat. Protoc..

[26] Myers K.J., Murthy S., Flanigan A., Witchell D.R., Butler M., Murray S., Siwkowski A., Goodfellow D., Madsen K., Baker B. (2003). Antisense oligonucleotide blockade of tumor necrosis factor-α in two murine models of colitis. J. Pharmacol. Exp. Ther..

[27] Naito Y., Takagi T., Uchiyama K., Kuroda M., Kokura S., Ichikawa H., Yanagisawa R., Inoue K., Takano H., Satoh M. (2004). Reduced intestinal inflammation induced by dextran sodium sulfate in interleukin-6-deficient mice. Int. J. Mol. Med..

[28] Atreya R., Zimmer M., Bartsch B., Waldner M.J., Atreya I., Neumann H., Hildner K., Hoffman A., Kiesslich R., Rink A.D. (2011). Antibodies against tumor necrosis factor (TNF) induce T-cell apoptosis in patients with inflammatory bowel diseases via TNF receptor 2 and intestinal CD14^+^ macrophages. Gastroenterology.

[29] Morita I. (2002). Distinct functions of COX-1 and COX-2. Prostaglandins Other Lipid Mediat..

[30] Wiercińska-Drapało A., Flisiak R., Prokopowicz D. (1999). Effects of ulcerative colitis activity on plasma and mucosal prostaglandin E_2_ concentration. Prostaglandins Other Lipid Mediat..

[31] Lauritsen K., Laursen L.S., Kjeldsen J., Bukhave K., Hansen T.K., Rask-Madsen J. (1995). Effects of mesalazine on the formation of lipoxygenase and cyclooxygenase products. Adv. Exp. Med. Biol..

[32] Perkins N.D., Gilmore T.D. (2006). Good cop, bad cop: The different faces of NF-κB. Cell Death Differ..

[33] Hayden M.S., Ghosh S. (2004). Signaling to NF-κB. Genes Dev..

[34] Lawrence T., Bebien M., Liu G.Y., Nizet V., Karin M. (2005). IKKα limits macrophage NF-κB activation and contributes to the resolution of inflammation. Nature.

[35] Fiore C., Eisenhut M., Ragazzi E., Zanchin G., Armanini D. (2005). A history of the therapeutic use of liquorice in Europe. J. Ethnopharmacol..

[36] Yoshida T., Abe K., Ikeda T., Matsushita T., Wake K., Sato T., Sato T., Inoue H. (2007). Inhibitory effect of glycyrrhizin on lipopolysaccharide and d-galactosamine-induced mouse liver injury. Eur. J. Pharmacol..

[37] Lin G., Nnane I.P., Cheng T.Y. (1999). The effects of pretreatment with glycyrrhizin and glycyrrhetinic aicd on the retrosine-induced hepatotoxicity in rats. Toxicon.

[38] Wang C.Y., Kao T.C., Lo W.H, Yen G.C. (2011). Glycyrrhizic acid and 18β-glyccyrrhetinic acid modulate lipopolysaccharide-induced inflammatory response by suppression of NF-κB through PI3K p110δ and p110γ inhibitions. J. Agric. Food Chem..

[39] Ukil A., Kar S., Srivastav S., Ghosh K., Das P.K. (2011). Curative effect of 18β-glyccyrrhetinic acid in experimental visceral leishmaniasis depends on phosphatase-dependent modulation of celluar MAP kinases. PLoS ONE.

[40] Hendrickson B.A., Gokhale R., Cho J.H. (2002). Clinical aspects and pathophysiology of inflammatory bowel disease. Clin. Microbiol. Rev..

[41] Murthy S.N., Cooper H.S., Shim H., Shah R.S., Ibrahim S.A., Sedergran D.J. (1993). Treatment of dextran sulfate sodium induced murine colitis by intracolonic cyclosporine. Dig. Dis. Sci..

[42] Kim S.J., Kim M.C., Um J.Y., Hong S.H. (2010). The beneficial effect of vanillic acid on ulcerative colitis. Molecules.

[43] Hamamoto N., Maemura K., Hirata I., Murano M., Sasaki S., Katsu K. (1999). Inhibition of dextran sulphate sodium (DSS)-induced colitis in mice by intracolonically administered antibodies against adhesion molecules (endothelial leucocyte adhesion molecule-1 (ELAM-1) or intercellular adhesion molecule-1 (ICAM-1)). Clin. Exp. Immunol..

